# Reevaluating Heart Transplant Compatibility: A Retrospective Analysis Using Echocardiographic Left Ventricular Mass Matching

**DOI:** 10.3390/jcm15155980

**Published:** 2026-07-31

**Authors:** Anthony Altobelli, Laura Parker, Deepa Iyer, Sai Somisetty, Maya Guglin

**Affiliations:** Rutgers Robert Wood Johnson Medical School, New Brunswick, NJ 08901, USA; aa3003@rwjms.rutgers.edu (A.A.IV); lrp87@rwjms.rutgers.edu (L.P.); deepa.iyer@rwjbh.org (D.I.); srs412@scarletmail.rutgers.edu (S.S.)

**Keywords:** heart transplantation, donor recipient size matching, predicted heart mass ratio, left ventricular mass, echocardiography, obesity, sex mismatch, organ allocation

## Abstract

**Background/Objectives:** Accurate size-matching between donor and recipient is central to adult heart transplantation, yet the optimal operational metric remains unsettled. We compared the predicted heart mass ratio (PHMr) with a ratio pairing measured donor left ventricular mass and predicted recipient left ventricular mass (mLVM:pLVM) to determine how each classifies the size acceptability of donor offers. **Methods:** In a retrospective single-center cohort of 594 adult donor offers evaluated for 22 recipients during 2023, offers were classified as acceptable under bilateral windows (PHMr 0.86 to 1.14, mLVM:pLVM 0.80 to 1.20) and under lower-bound-only thresholds. Recipient-clustered models with cluster-robust standard errors were the primary analysis, with a generalized estimating equation and a linear mixed model as sensitivity analyses. Prespecified subgroups were defined by sex and body mass index. **Results:** Under bilateral windows, PHMr classified more offers as acceptable than mLVM:pLVM (46.3% versus 40.1%), but the overall difference was not significant in the primary analysis (cluster-robust risk difference 6.2 percentage points, 95% CI −0.5 to 13.0, *p* = 0.068) and reached significance only in sensitivity analyses. Agreement was low (Cohen’s κ 0.12 to 0.24). The between-method difference varied by donor sex, confirmed by interaction testing: PHMr classified more male donor offers as acceptable, whereas mLVM:pLVM classified more female donor offers as acceptable. Under lower-bound-only thresholds, significant differences were confined to donor sex. **Conclusions:** Metric and threshold choice changed which offers were classified as acceptable. These differences reflect operational discordance rather than clinical superiority and warrant validation linked to outcomes.

## 1. Introduction

Heart transplantation is a lifesaving therapy for end stage heart failure. In 2023, the volume of heart transplantation in the United States reached a record 4599 procedures, of which 4092 were performed in adults, a 101.1% increase since 2012 [1]. Compatibility between a donor and a candidate depends on several clinical and logistical factors, including age, ABO blood group, ischemic time, and recipient urgency and comorbidity burden [2]. Heart size-matching is one core determinant within this evaluation.

Historically, donor body weight served as the default surrogate for cardiac size under guidance from the International Society for Heart and Lung Transplantation (ISHLT), with donors up to 30% below recipient weight generally considered acceptable and additional caution advised for female donor to male recipient pairings when donor weight was more than 20% lower [3]. Over the past decade, predicted heart mass (PHM) has been reported to capture anatomic and physiologic size more accurately than body weight and to predict outcomes related to sex mismatch more accurately [4]. Under-sizing by PHM was associated with higher rates of primary graft dysfunction (PGD) [5] and with reduced early survival. In a registry analysis, severe under-sizing by PHM carried a hazard ratio for mortality at 1 year of 1.34 (95% CI 1.13 to 1.59) [6]. These data support PHM as the current standard for adult size-matching [6,7].

Different size metrics do not classify the same offer identically, and this discordance is common. An unacceptable size match is a frequent reason for donor heart turndown and accounts for roughly 15% of declined offers in national registry data [6]. The choice of metric changes that judgment. Among offers declined for donor size or weight, about one third (32%) met the PHM threshold for an adequate size match and could plausibly have been accepted [6]. This discordance between weight and PHM is strongly directional. The fraction of size-based turndowns that were acceptable by PHM ranged from 17% for female donor to male recipient offers to 98% for male donor to female recipient offers [4,6]. Because under-sizing tracks with PGD and mortality, the metric used to screen size can change which offers are declined and, potentially, the outcomes that follow [5,6].

Reliance on anthropometric surrogates introduces additional misclassification in obesity, where predicted mass can diverge from true myocardial (fat free) mass [8,9,10]. Higher recipient body mass index (BMI) was associated with progressively larger discrepancies in transplanted heart mass and with worse outcomes at the extremes [10]. To address this limitation, an alternative strategy uses echocardiographically measured left ventricular mass (LVM) to form a ratio of donor-to-recipient mass. In the prospective multicenter Donor Heart Study, a ratio based on measured LVM was associated with outcomes in a pattern in which risk rose at low ratios below 0.80 and at high ratios above 1.20, and it predicted outcomes better than modeled approaches in recipients with obesity [11,12].

Against this background, we compared the proportion of donor offers classified as acceptable by the current standard PHM method with the proportion classified as acceptable by an mLVM method in a contemporary single-center cohort. We prespecified bilateral acceptance windows (PHMr 0.86 to 1.14, mLVM:pLVM 0.80 to 1.20) and a lower-bound-only framework, and we evaluated how the design of the acceptance range influenced classification across subgroups defined by sex and BMI.

## 2. Materials and Methods

### 2.1. Study Design

**Figure 1:**
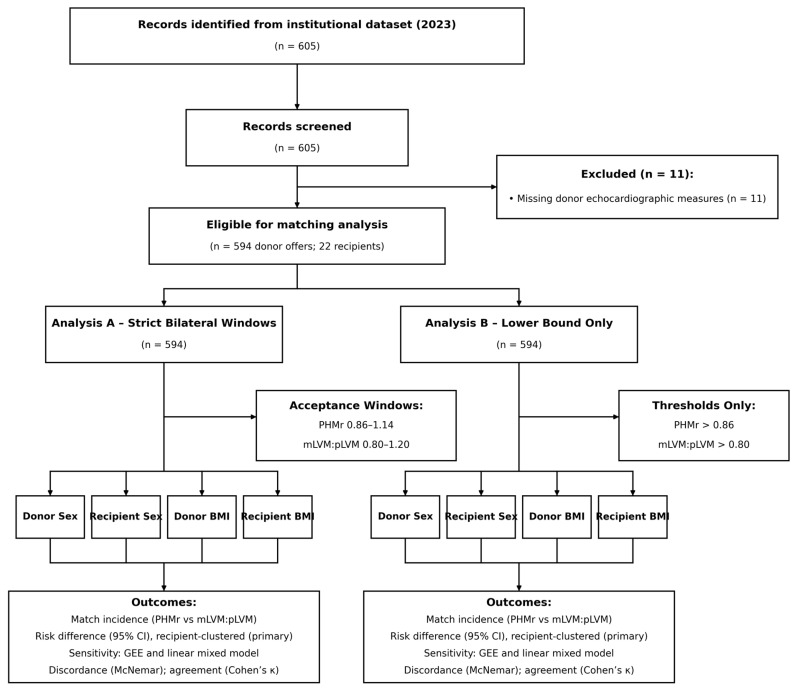
Study flow and analytic design. Donor offers evaluated at the study center in 2023 were screened; 11 offers with missing donor echocardiographic measures were excluded, yielding an analytic cohort of 594 donor offers evaluated for 22 recipients. Size acceptability was assessed under two frameworks applied to the same offers: (A) strict bilateral acceptance windows (PHMr 0.86–1.14; mLVM:pLVM 0.80–1.20) and (B) lower-bound-only thresholds (PHMr ≥ 0.86; mLVM:pLVM ≥ 0.80). Within each framework, analyses were repeated across prespecified subgroups defined by donor sex, recipient sex, donor BMI, and recipient BMI (<35 vs. ≥35 kg/m^2^). Because offers were nested within recipients, between-method comparisons used a recipient-clustered (cluster-robust) linear model as the primary analysis, with generalized estimating equation (GEE) and linear mixed-model sensitivity analyses, alongside raw match incidence, offer-level McNemar testing of discordance, and Cohen’s κ for agreement. Abbreviations: BMI, body mass index; CI, confidence interval; GEE, generalized estimating equation; mLVM:pLVM, measured donor left ventricular mass to predicted recipient left ventricular mass ratio; PHMr, predicted heart mass ratio; RD, risk difference.

This retrospective cohort study included all adult donor recipient heart offer evaluations conducted at Robert Wood Johnson University Hospital Transplant Center in New Brunswick, NJ, USA, from 1 January 2023 to 31 December 2023. No patients were prospectively recruited. The cohort comprised adult heart transplant candidates receiving care at the center and donor offers identified through Organ Offer Reports in the United Network for Organ Sharing (UNOS) DonorNet portal. Donor clinical data were obtained from UNOS records, and recipient clinical data were obtained from the institutional electronic medical record (Figure 1).

### 2.2. Data Collection and Variables

For both donors and recipients, we collected demographic data, including age, sex, height, weight, BMI, body surface area (BSA), and ABO blood type. For donors, we extracted the left ventricular internal diameter at end diastole (LVIDd), the posterior wall thickness at end diastole (PWT), and the interventricular septal thickness at end diastole (IVSd) from echocardiography reports. We calculated measured left ventricular mass (mLVM) with the American Society of Echocardiography and European Association of Cardiovascular Imaging (ASE/EACVI) formula: mLVM (g) = 0.8 × (1.04 × [(LVIDd + IVSd + PWT)^3^ − LVIDd^3^]) + 0.6 [13]. We estimated predicted left ventricular mass (pLVM) for donors and recipients with the Multi-Ethnic Study of Atherosclerosis equation: pLVM (g) = a × height^0.54 (m) × weight^0.61 (kg), where a = 6.82 for women and 8.25 for men [14].

The predicted heart mass ratio (PHMr), calculated from the predicted left and right ventricular masses of the donor and recipient, has been described previously in detail [4,6,7]. For the strategy based on mLVM, the donor component was derived from echocardiography (mLVM), whereas the recipient left ventricular mass was predicted (pLVM) because the native recipient left ventricle is diseased. The mLVM ratio in this analysis therefore reflects measured donor LV mass relative to predicted recipient LV mass. ISHLT recommends that the donor-to-recipient predicted heart mass ratio remain within 0.86 to 1.14 for optimal size-matching [7]. We applied 0.86 ≤ PHMr ≤ 1.14 and 0.80 ≤ mLVM:pLVM ≤ 1.20 as prespecified bilateral acceptance windows and evaluated lower-bound-only thresholds (PHMr ≥ 0.86, mLVM:pLVM ≥ 0.80) in sensitivity analyses.

### 2.3. Outcomes

We evaluated PHMr and mLVM:pLVM under a bilateral window framework, in which both bounds were applied, and under a lower-bound-only threshold, in which only the lower bound was applied. The primary descriptive outcome was match incidence for each strategy under the prespecified acceptance criteria. We summarized discordance between strategies as the number of offers accepted by one method but not the other, and we quantified agreement between strategies within each framework.

### 2.4. Statistical Analysis

We summarized match incidence for each strategy as the count and proportion of offers classified as size acceptable. Because both metrics were applied to the same offers, we compared paired classifications rather than independent proportions.

At the offer level, we compared match incidence between strategies with the McNemar test and estimated paired risk differences with 95% CIs. This offer-level analysis treated each offer as an independent unit. We quantified chance-corrected agreement between the two classifications with Cohen’s κ and estimated 95% CIs for κ by bootstrap resampling of recipient clusters with 2000 replications.

The 594 offers were not independent, because they were nested within 22 unique recipients, and each recipient could be evaluated against multiple donor offers. To account for this clustering, we specified recipient-level models as the primary comparison between methods. We modeled size acceptability as a linear function of matching strategy, so that the model coefficient estimated the risk difference between methods directly. We estimated standard errors with a cluster-robust (sandwich) estimator that used the recipient as the clustering unit. Because inference rested on only 22 recipient clusters, we applied the CR1 small sample adjustment and referred test statistics to a t distribution with G minus 1 degrees of freedom, where G is the number of recipient clusters contributing to a given estimate. For the overall comparison this value was 21, and for each subgroup it equaled the number of recipients in that stratum minus one. To guard against anticonservative inference when the number of clusters is small, we additionally computed, for the overall comparison, bias-reduced CR2 standard errors with Satterthwaite degrees of freedom and a wild cluster bootstrap using Rademacher weights. We report the cluster-robust CR1 risk difference, its 95% CI, and its *p* value as the primary result.

We conducted two further analyses to assess the stability of the primary findings under alternative modeling assumptions. First, we fitted a generalized estimating equation (GEE) with an identity link and an exchangeable working correlation structure, clustered on recipient. Second, we fitted a linear mixed model with a random intercept for the recipient. Both models estimated the risk difference between methods, which we report with its 95% CI and *p* value.

We repeated all analyses within prespecified subgroups defined by donor sex, recipient sex, recipient BMI (<35 versus ≥35 kg/m^2^), and donor BMI (<35 versus ≥35 kg/m^2^), under both the bilateral windows and the lower-bound-only thresholds. Strata that contained too few recipients to support stable estimation were not modeled in the clustered analyses. As exploratory analyses, we tested effect modification of the between-method risk difference by donor sex and by recipient BMI category using cluster-robust linear models that included an interaction term between method and subgroup. These interaction tests were not adjusted for multiplicity, and subgroup *p* values should be read as exploratory. A two-sided *p* value below 0.05 defined statistical significance. All analyses were performed in Python version 3.11 with statsmodels, pandas, NumPy, and SciPy packages.

### 2.5. Missing Data

We excluded records from the analytic cohort only when required measurements were unavailable, specifically the donor echocardiographic measures needed to calculate mLVM, as described above (n = 11). We performed no additional imputation. The 594 offers that formed the analytic cohort were complete for all analyzed variables.

### 2.6. Ethical Oversight and Organ Sourcing

This retrospective analysis of de-identified data was approved by the Rutgers Institutional Review Board (Protocol Pro2025001543, approved 3 October 2025) with a waiver of informed consent. Organs referenced in this dataset were procured in accordance with the World Health Organization (WHO) Guiding Principles on Human Cell, Tissue, and Organ Transplantation. Autonomous consent free from coercion was obtained from donors or their next of kin, and no organs or tissues were sourced from executed prisoners or prisoners of conscience.

### 2.7. Use of Generative AI

During the preparation of this manuscript, a generative AI assistant (Claude, Anthropic; model Claude Opus 4.8) was used to assist with revising manuscript text and preparing the figures and the concordance/discordance table. All statistical analyses were designed and conducted by the study team, and all data, reported values, and figures were verified by the authors against the source dataset and analytic output. The authors take full responsibility for the accuracy and integrity of the content.

## 3. Results

In 2023, we identified 605 records. We excluded 11 donors who lacked echocardiographic measures, which yielded 594 donor offers with complete PHMr and mLVM:pLVM data (Table 1). These 594 offers were nested within 22 unique recipients. Because repeated offers to the same recipient are not independent, we report offer-level estimates together with recipient-clustered estimates, and we treat the cluster-robust linear model as the primary comparison between methods, with the GEE and the linear mixed model as sensitivity analyses.

### 3.1. Overall Match Incidence Under Strict Bilateral Windows

Under bilateral windows, PHMr classified 46.3% of offers as acceptable (275/594) compared with 40.1% for mLVM:pLVM (238/594). The offer-level risk difference (RD) was 6.2 percentage points (pp) (95% CI 0.6 to 11.9; McNemar *p* = 0.025). After accounting for clustering within recipients, the cluster-robust RD was 6.2 pp with wider bounds that crossed the null (95% CI −0.5 to 13.0; *p* = 0.068; 21 degrees of freedom). The bias-reduced CR2 estimate and the wild cluster bootstrap were concordant (*p* = 0.072 and *p* = 0.089). The sensitivity analyses were directionally consistent and reached significance, with a GEE estimate of 6.2 pp (95% CI 0.0 to 12.4; *p* = 0.049) and a mixed-model estimate of 6.2 pp (95% CI 0.6 to 11.8; *p* = 0.029).

### 3.2. Sex-Stratified Match Incidence Under Strict Bilateral Windows

The largest between-method difference occurred among male donor offers. Among male donor offers (n = 252), PHMr classified 69.0% as acceptable (174/252) compared with 43.7% (110/252), an offer-level RD of 25.4 pp (95% CI 17.0 to 33.8; McNemar *p* < 0.001). The cluster-robust estimate was 25.4 pp (95% CI 12.7 to 38.1; *p* < 0.001) and was concordant with the GEE estimate (25.4 pp, 95% CI 13.9 to 36.8; *p* < 0.001) and the mixed-model estimate (25.4 pp, 95% CI 17.1 to 33.7; *p* < 0.001). Among female donor offers (n = 342), the direction reversed and PHMr classified fewer offers as acceptable, with PHMr at 29.5% (101/342) and mLVM:pLVM at 37.4% (128/342), an offer-level RD of −7.9 pp (95% CI −14.9 to −0.8; McNemar *p* = 0.023). The cluster-robust estimate was −7.9 pp with bounds that crossed the null (95% CI −19.3 to 3.5; *p* = 0.17). The mixed-model estimate was statistically significant (−7.9 pp, 95% CI −14.8 to −1.0; *p* = 0.025), whereas the GEE estimate was not (−7.9 pp, 95% CI −18.4 to 2.6; *p* = 0.14).

Among male recipient offers (n = 574), PHMr classified 46.3% as acceptable (266/574) compared with 39.9% (229/574), an offer-level RD of 6.4 pp (95% CI 0.7 to 12.2; McNemar *p* = 0.023). The cluster-robust RD was 6.4 pp with bounds that crossed the null (95% CI −0.5 to 13.4; *p* = 0.069), while the GEE (6.4 pp, 95% CI 0.1 to 12.8; *p* = 0.047) and mixed-model (6.4 pp, 95% CI 0.8 to 12.1; *p* = 0.026) estimates were statistically significant. Among female recipient offers (n = 20), both methods classified 45.0% as acceptable (9/20), an offer-level RD of 0 pp (95% CI −30.8 to 30.8; McNemar *p* = 0.68). The clustered models did not estimate this stratum because it contained only two recipients.

### 3.3. BMI Stratified Match Incidence Under Strict Bilateral Windows

Among recipients with BMI < 35 kg/m^2^ (n = 495 offers), PHMr classified 49.5% as acceptable (245/495) compared with 39.8% (197/495), an offer-level RD of 9.7 pp (95% CI 3.5 to 15.9; McNemar *p* = 0.001) and a cluster-robust RD of 9.7 pp (95% CI 4.2 to 15.2; *p* = 0.002). The GEE (9.7 pp, 95% CI 4.7 to 14.7; *p* < 0.001) and mixed-model (9.7 pp, 95% CI 3.5 to 15.8; *p* = 0.002) estimates agreed. Among recipients with BMI ≥ 35 kg/m^2^ (n = 99), the methods did not differ significantly, with 30.3% (30/99) compared with 41.4% (41/99), an offer-level RD of −11.1 pp (95% CI −24.4 to 2.2; McNemar *p* = 0.14) and a cluster-robust RD of −11.1 pp (95% CI −34.7 to 12.5; *p* = 0.23). This stratum rested on only three recipients and warrants caution.

Among donors with BMI < 35 kg/m^2^ (n = 448), PHMr classified 46.0% as acceptable (206/448) compared with 39.5% (177/448), an offer-level RD of 6.5 pp (95% CI 0.0 to 12.9; McNemar *p* = 0.043) and a cluster-robust RD of 6.5 pp (95% CI 0.6 to 12.4; *p* = 0.033). The GEE (*p* = 0.018) and mixed-model (*p* = 0.049) estimates agreed. Among donors with BMI ≥ 35 kg/m^2^ (n = 146), the difference did not reach statistical significance, with 47.3% (69/146) compared with 41.8% (61/146), an offer-level RD of 5.5 pp (95% CI −5.9 to 16.9; McNemar *p* = 0.39) and a cluster-robust RD of 5.5 pp (95% CI −9.8 to 20.8; *p* = 0.46). Across the bilateral window strata, the GEE and mixed-model estimates matched the cluster-robust results in magnitude and direction.

### 3.4. Sensitivity Analysis: Lower-Bound-Only Windows

Removing the upper bound narrowed the between-method differences overall and confined the significant differences to donor sex. Overall, PHMr classified 52.2% as acceptable (310/594) compared with 54.4% (323/594), an offer-level RD of −2.2 pp (95% CI −7.9 to 3.5; McNemar *p* = 0.42) and a cluster-robust RD of −2.2 pp (95% CI −8.7 to 4.3; *p* = 0.49).

Among male donor offers (n = 252), PHMr classified 81.3% as acceptable (205/252) compared with 61.9% (156/252), an offer-level RD of 19.4 pp (95% CI 11.8 to 27.1; McNemar *p* < 0.001) and a cluster-robust RD of 19.4 pp (95% CI 6.7 to 32.2; *p* = 0.005). Among female donor offers (n = 342), the direction reversed, with PHMr at 30.7% (105/342) and mLVM:pLVM at 48.8% (167/342), an offer-level RD of −18.1 pp (95% CI −25.3 to −10.9; McNemar *p* < 0.001) and a cluster-robust RD of −18.1 pp (95% CI −26.7 to −9.5; *p* < 0.001). Among recipients with BMI ≥ 35 kg/m^2^ (n = 99), PHMr classified 30.3% as acceptable (30/99) compared with 47.5% (47/99). The offer-level difference was statistically significant (RD −17.2 pp, 95% CI −30.5 to −3.8; McNemar *p* = 0.015), as were the GEE (*p* = 0.001) and mixed-model (*p* = 0.012) estimates, but the primary cluster-robust estimate was not (RD −17.2 pp, 95% CI −37.0 to 2.6; *p* = 0.070), and this stratum rested on only three recipients. No other stratum reached significance under the primary clustered analysis. The GEE and mixed-model estimates were otherwise concordant.

### 3.5. Formal Interaction Tests

**Figure 2:**
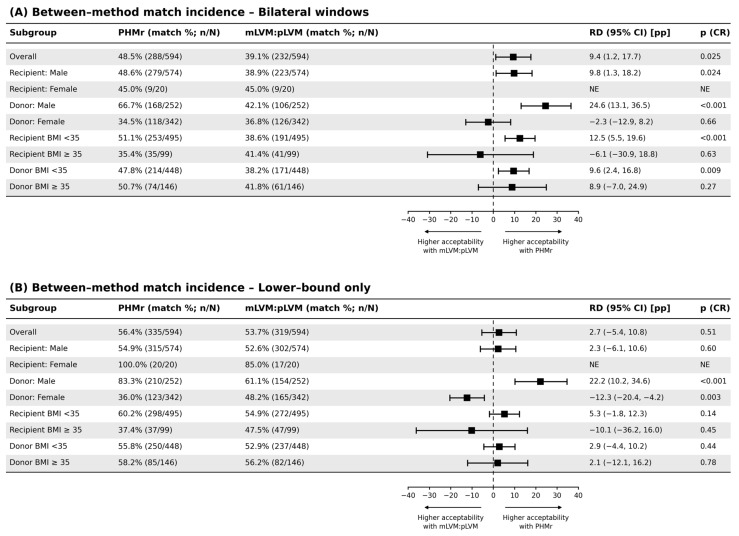
Between-method match incidence and risk differences by acceptance framework and prespecified subgroup. Donor offer size acceptability was classified by the predicted heart mass ratio (PHMr) versus the measured donor left ventricular mass to predicted recipient left ventricular mass ratio (mLVM:pLVM) under (**A**) bilateral acceptance windows (PHMr 0.86 to 1.14; mLVM:pLVM 0.80 to 1.20) and (**B**) lower-bound-only thresholds (PHMr ≥ 0.86; mLVM:pLVM ≥ 0.80). For each subgroup, raw match rates are given as the percentage of offers classified as acceptable (n/N) by each metric. Squares denote the recipient-clustered (cluster-robust) between-method risk difference (RD, percentage points, pp) and horizontal lines denote the 95% confidence interval (CI). The dashed vertical line marks no difference (RD = 0). Positive values indicate higher acceptability with PHMr and negative values indicate higher acceptability with mLVM:pLVM. The tabulated RD (95% CI) and *p* values are the cluster-robust estimates. NE: not estimable in the clustered model (female recipients, 2 recipients). The double dagger marks the recipient BMI ≥ 35 kg/m^2^ stratum, which rests on 3 recipients and should be interpreted with caution. Abbreviations: BMI, body mass index; CI, confidence interval; CR, cluster robust; mLVM:pLVM, measured donor left ventricular mass to predicted recipient left ventricular mass ratio; PHMr, predicted heart mass ratio; pp, percentage points; RD, risk difference.

Formal interaction tests corroborated the subgroup patterns. Under bilateral windows, the between-method risk difference differed by donor sex (difference in RD 33.3 pp; *p* = 0.001) and by recipient BMI category (difference in RD −20.8 pp for BMI ≥ 35 versus <35 kg/m^2^; *p* = 0.007). Under lower-bound-only thresholds, the difference by donor sex was again present (difference in RD 37.6 pp; *p* < 0.001), as was the difference by recipient BMI category (−18.0 pp; *p* = 0.008). These interaction tests were prespecified as exploratory and were not adjusted for multiplicity (Figure 2, Table 2).

## 4. Discussion

### 4.1. Principal Findings

In this single-center cohort of 594 donor offers evaluated for 22 adult recipients over one year, the choice of size-matching metric changed which donors were classified as size acceptable. Under the prespecified bilateral windows, PHMr classified a higher proportion of offers as acceptable overall than the mLVM:pLVM ratio derived from echocardiography, although this overall difference did not reach statistical significance in the primary recipient-clustered model (RD 6.2 pp, 95% CI −0.5 to 13.0, *p* = 0.068) and reached significance only in the GEE and mixed-model sensitivity analyses. Agreement between the two classifications was low, and discordance was directional, favoring offers acceptable to PHMr alone. Because repeated offers cluster within recipients, we treated the recipient-clustered estimates as primary. Clustering widened the confidence intervals and attenuated the overall difference to borderline significance. The between-method difference nonetheless remained statistically significant within specific strata across all models, most consistently among male donor offers and among recipients with BMI < 35 kg/m^2^. When only the lower bound was applied, the between-method differences narrowed overall and the significant differences were confined to donor sex, with a smaller offer-level difference among recipients with BMI ≥ 35 kg/m^2^. The change between the two frameworks shows that the design of the acceptance window influenced which offers were classified as acceptable.

These findings describe classification behavior rather than clinical benefit. Without posttransplant outcomes, we cannot determine whether offers acceptable to PHMr alone represent safe expansion of the donor pool or higher risk acceptances that an approach based on mLVM would exclude. The observed differences therefore describe how selection policies might shift the acceptability of donor offers under alternative size-matching strategies. They are not evidence that one strategy is clinically superior.

### 4.2. Positioning Within the Literature

Prior work has established predicted heart mass as a practical alternative to weight-based matching that is grounded in cardiac physiology. Undersizing by PHM was associated with higher rates of primary graft dysfunction and worse early survival in registry analyses [4,5,6]. Our results are consistent with this literature. PHMr, constrained by symmetric bounds, classified more offers as acceptable than mLVM:pLVM in several strata, particularly among male donors and recipients with BMI < 35 kg/m^2^.

The two metrics diverged in opposite directions by donor sex. Among male donor offers, PHMr classified more offers as acceptable than mLVM:pLVM (bilateral RD 25.4 pp, lower-bound RD 19.4 pp, both statistically significant across models). Among female donor offers, mLVM:pLVM classified more offers as acceptable (bilateral RD −7.9 pp, statistically significant at the offer level and in the mixed model; lower-bound RD −18.1 pp, statistically significant across all models). Formal interaction testing showed that the between-method difference differed by donor sex (difference in RD 33.3 pp under bilateral windows, *p* = 0.001; 37.6 pp under lower-bound-only thresholds, *p* < 0.001) and by recipient BMI category (−20.8 pp, *p* = 0.007; and −18.0 pp, *p* = 0.008), although these tests were exploratory and were not adjusted for multiplicity. This divergence is consistent with the recognized tendency of predicted mass metrics to differ from measured myocardial mass by sex and at the extremes of body size [4,6].

Imaging-derived myocardial mass is especially informative at the extremes of body habitus. In the Donor Heart Study, a donor-to-recipient ratio based on measured LVM was associated with death or retransplantation in a pattern in which risk was elevated below a ratio of 0.80 and above 1.20, and it predicted outcomes better than modeled measures in recipients with obesity [12]. Our mLVM ratio pairs measured donor LV mass with predicted recipient LV mass, because the recipient left ventricle is diseased. This measured-to-predicted construct differs from a measured-to-measured comparison and may limit generalizability to cohorts that define recipient mass differently.

The sex-stratified patterns warrant caution. Female recipients were few, and the clustered models could not estimate the female recipient stratum. The donor sex and recipient sex findings are best regarded as hypothesis-generating. Larger multicenter cohorts with linkage to outcomes are needed to determine whether sex modifies the association between these metrics and clinical endpoints.

### 4.3. Discordance and Operational Implications

The low agreement between PHMr and mLVM:pLVM shows that the two classifications are not interchangeable. Under bilateral windows, chance-corrected agreement was slight (Cohen’s κ 0.12, 95% CI 0.06 to 0.18), and under lower-bound-only thresholds it was fair (κ 0.24, 95% CI 0.15 to 0.33). The confidence intervals for the overall comparisons excluded zero, so agreement exceeded chance, but it remained well below the range that would indicate the metrics are interchangeable. Under bilateral windows, the metrics disagreed on 257 of 594 offers (43.3%), and this discordance favored acceptability by PHMr alone, with 147 offers acceptable to PHMr only compared with 110 acceptable to mLVM:pLVM only. Adopting one metric in place of the other would change the overall proportion of offers deemed acceptable and which specific offers pass initial screening. Such a change could affect acceptance patterns and waiting time. The direction and clinical value of any such shift cannot be inferred without outcome data and without accounting for the donor quality and recipient acuity variables that routinely inform organ selection.

### 4.4. Clinical Interpretation

Within the limits of a study confined to classification, PHMr with bilateral windows may serve as a pragmatic default for initial size screening, pending validation that links classification differences to posttransplant outcomes. In recipients with obesity, in borderline offers, or when the two metrics disagree, incorporating measured donor LV mass as a complementary input is reasonable when echocardiographic data are available, because prior evidence linked to outcomes argues against extreme mLVM ratios [12].

Size-matching is only one component of donor selection and must be weighed against recipient hemodynamics and clinical urgency. Pulmonary hypertension or elevated pulmonary vascular resistance, durable left ventricular assist device (LVAD) support, and high acuity status may justify deliberate over-sizing and greater avoidance of under-sizing. Donor age may also modify tolerance for modest under-sizing, and younger donors are often considered more acceptable in sex-mismatched pairings. We did not model these factors, and future validation should incorporate them explicitly.

### 4.5. Study Limitations

This study has several limitations. Its single-center design, its window of one year, and its small number of unique recipients constrain generalizability. Because 594 offers were evaluated across only 22 recipients, the offers were not independent. We addressed this with recipient-clustered models, but the limited number of clusters reduces precision, as reflected in the wide confidence intervals for sparsely represented strata, for example recipients with BMI ≥ 35, which comprised three recipients. The analysis evaluated the classification of size match rather than clinical outcomes such as primary graft dysfunction, right heart failure, early mortality, or survival at one year. Several strata, particularly female recipients and the joint sex and BMI subgroups, were underpowered and should not support policy-level inference. Finally, we did not account for determinants of donor acceptance beyond size, including recipient pulmonary pressures, donor age and quality, durable LVAD support, and recipient acuity, each of which can influence decisions about over-sizing and under-sizing.

### 4.6. Future Directions

Several directions follow from this work. First, future studies should link classification by PHMr and by mLVM to posttransplant outcomes in multicenter cohorts, with prespecified evaluation of recipients with obesity and of sex-mismatched pairs. Second, they should incorporate recipient hemodynamics, durable LVAD status, and acuity into models that reflect how size-matching is applied in practice. Third, they should test whether symmetric or asymmetric acceptance windows better calibrate risk, including approaches that model the elevated risk reported at both low and high mLVM ratios. Pragmatic implementation studies should also assess the feasibility of routine extraction of donor echocardiographic measures, interobserver reproducibility, the effect on acceptance rates and waiting times, and equity across strata defined by sex and body size.

## 5. Conclusions

In this single-center retrospective analysis of adult donor offers, PHMr and an mLVM:pLVM ratio informed by echocardiography produced different classifications of size acceptability across common thresholding strategies. Under bilateral windows, PHMr classified a higher proportion of offers as acceptable overall. This overall difference was attenuated to borderline, non-significant under the primary recipient-clustered model, but the between-method difference remained statistically significant within specific strata, in particular among male donors and among recipients with BMI < 35 kg/m^2^. Agreement between the metrics was low, and discordance was concentrated in offers acceptable to PHMr alone. These stratum-specific differences persisted after accounting for clustering within recipients. They reflect operational discordance between classification methods rather than clinical superiority, because we did not evaluate posttransplant outcomes and because acceptance decisions also depend on recipient hemodynamics, mechanical circulatory support, and acuity. The choice of size-matching metric and threshold can change the triage of donor offers. These results support multicenter validation linked to outcomes to determine whether discordant classifications represent safe expansion of the donor pool or clinically consequential misclassification, in particular in underpowered subgroups and borderline offers.

## Figures and Tables

**Table 1:** Baseline characteristics of donors and intended recipients. Summary of demographic and anthropometric features for donors (N = 594) and intended recipients (N = 22) included in the analytic cohort. Continuous variables are reported as mean ± SD and median (IQR), as appropriate; categorical variables are reported as number (percent). Body mass index (BMI) is calculated as weight (kg)/height (m)^2. Body surface area (BSA) is calculated using standard formulae. Predicted heart mass (PHM) and predicted left ventricular mass (pLVM) were derived from published sex-specific equations; measured LV mass was obtained by echocardiography when available. Missingness is reported per variable. Abbreviations: BMI, body mass index; BSA, body surface area; PHM, predicted heart mass; pLVM, predicted LV mass; LV, left ventricle.

Characteristic	Donors (N = 594)	Recipients (N = 22)
Demographics		
Age, years	39 ± 9.3	48.3 ± 11.6
	39 [32–40]	48 [41–51]
Sex, male—n (%)	252 (42.4%)	20 (90.9%)
Sex, female—n (%)	342 (57.6%)	2 (9.1%)
Anthropometrics		
Height, cm	167.2 ± 9.7	174.5 ± 11.7
Weight, kg	84.9 ± 19.2	90.3 ± 19.5
Body Mass Index, kg/m^2^	30.5 ± 7.2	29.4 ± 5.3
BMI < 35—n (%)	448 (75.4%)	19 (86.4%)
BMI ≥ 35—n (%)	146 (24.6%)	3 (13.6%)
Body Surface Area, m^2^	1.97 ± 0.23	2.06 ± 0.26
Blood type		
ABO A—n (%)	133 (22.4%)	4 (18.2%)
ABO B—n (%)	76 (12.8%)	3 (13.6%)
ABO AB—n (%)	6 (1.0%)	1 (4.5%)
ABO O—n (%)	379 (63.8%)	14 (63.6%)
Cardiac size metrics		
Predicted heart mass (PHM), g	171.3 ± 28.9	196.4 ± 33.7
Predicted LV mass (pLVM), g	134.5 ± 19.1	158.1 ± 24.4
Measured LV mass (echo), g	151.8 ± 57.3	—
Missing Data		
Any missing data—n (%)	0 (0.0%)	0 (0.0%)

Note: Continuous variables are mean ± SD; [Median (IQR)].

**Table 2:** Concordance and discordance between PHMr and mLVM:pLVM size acceptability classifications, by acceptance framework and by donor sex. Each donor offer was classified as size acceptable or not by both metrics, which yields four paired categories: both acceptable, PHMr only, mLVM:pLVM only, and neither acceptable. Values are counts (row percent) within each stratum. The categories both and neither represent concordant classifications, whereas PHMr only and mLVM:pLVM only represent discordance. Cohen’s κ quantifies chance-corrected agreement between metrics, with the 95% CI obtained by bootstrap resampling of 22 recipient clusters with 2000 replications. The qualitative descriptor follows the Landis and Koch convention. The McNemar *p* value tests paired discordance between the two metrics. Abbreviations: CI, confidence interval; mLVM:pLVM, measured donor left ventricular mass to predicted recipient left ventricular mass ratio; PHMr, predicted heart mass ratio; κ, Cohen’s kappa.

Bilateral Acceptance Windows (PHMr 0.86–1.14; mLVM:pLVM 0.80–1.20)							
**Subgroup**	**n**	**Both Acceptable**	**PHMr Only**	**mLVM:pLVM Only**	**Neither**	**κ**	**McNemar *p***
Overall	594	128 (21.5)	147 (24.7)	110 (18.5)	209 (35.2)	0.12	0.025
Donor male	252	79 (31.3)	95 (37.7)	31 (12.3)	47 (18.7)	0.05	<0.001
Donor female	342	49 (14.3)	52 (15.2)	79 (23.1)	162 (47.4)	0.15	0.023
Lower-Bound-Only Thresholds (PHMr ≥ 0.86; mLVM:pLVM ≥0.80)							
**Subgroup**	**n**	**Both Acceptable**	**PHMr Only**	**mLVM:pLVM Only**	**Neither**	**κ**	**McNemar *p***
Overall	594	204 (34.3)	106 (17.8)	119 (20.0)	165 (27.8)	0.24	0.42
Donor male	252	132 (52.4)	73 (29.0)	24 (9.5)	23 (9.1)	0.10	<0.001
Donor female	342	72 (21.1)	33 (9.6)	95 (27.8)	142 (41.5)	0.25	<0.001

## Data Availability

The UNOS DonorNet Organ Offer Reports accessed for the purposes of this investigation are not open to the public. UNOS provides aggregated national, regional, state, and center-specific data as well as interactive dashboards and annual reports through the Organ Procurement and Transplantation Network website. De-identified data on the donors and recipients collected can be provided upon request to the corresponding author.

## Abbreviations

The following abbreviations are used in this manuscript:

BMI	Body Mass Index
BSA	Body Surface Area
CI	Confidence Interval
GEE	Generalized Estimating Equation
ISHLT	International Society for Heart and Lung Transplantation
IVSd	Interventricular Septal Thickness at End-Diastole
LV	Left Ventricle
LVAD	Left Ventricular Assist Device
LVIDd	Left Ventricular Internal Dimension at End-Diastole
LVM	Left Ventricular Mass
mLVM	Measured Left Ventricular Mass
mLVM:pLVM	Measured Donor Left Ventricular Mass to Predicted Recipient Left Ventricular Mass Ratio
PGD	Primary Graft Dysfunction
PHM	Predicted Heart Mass
PHMr	Predicted Heart Mass Ratio
pLVM	Predicted Left Ventricular Mass
pp	Percentage Points
PWT	Posterior Wall Thickness
RD	Risk Difference
SD	Standard Deviation
UNOS	United Network for Organ Sharing
WHO	World Health Organization

## References

[1] Colvin M.M., Smith J.M., Ahn Y.S., Lindblad K.A., Handarova D., Israni A.K., Snyder J.J. (2025). OPTN/SRTR 2023 Annual Data Report: Heart. Am. J. Transplant..

[2] Peled Y., Ducharme A., Kittleson M., Bansal N., Stehlik J., Amdani S., Saeed D., Cheng R., Clarke B., Dobbels F. (2024). International Society for Heart and Lung Transplantation Guidelines for the Evaluation and Care of Cardiac Transplant Candidates-2024. J. Heart Lung Transplant..

[3] Costanzo M.R., Dipchand A., Starling R., Anderson A., Chan M., Desai S., Fedson S., Fisher P., Gonzales-Stawinski G., Martinelli L. (2010). International Society of Heart and Lung Transplantation Guidelines. The International Society of Heart and Lung Transplantation Guidelines for the care of heart transplant recipients. J. Heart Lung Transplant..

[4] Reed R.M., Netzer G., Hunsicker L., Mitchell B.D., Rajagopal K., Scharf S., Eberlein M. (2014). Cardiac size and sex-matching in heart transplantation: Size matters in matters of sex and the heart. JACC Heart Fail..

[5] Gong T.A., Joseph S.M., Lima B., Gonzalez-Stawinski G.V., Jamil A.K., Felius J., Qin H., Saracino G., Rafael A.E., Kale P. (2018). Donor predicted heart mass as predictor of primary graft dysfunction. J. Heart Lung Transplant..

[6] Kransdorf E.P., Kittleson M.M., Benck L.R., Patel J.K., Chung J.S., Esmailian F., Kearney B.L., Chang D.H., Ramzy D., Czer L.S. (2019). Predicted heart mass is the optimal metric for size match in heart transplantation. J. Heart Lung Transplant..

[7] Khush K.K., Cherikh W.S., Chambers D.C., Harhay M.O., Hayes D., Hsich E., Meiser B., Potena L., Robinson A., Rossano J.W. (2019). The International Thoracic Organ Transplant Registry of the International Society for Heart and Lung Transplantation: Thirty-sixth adult heart transplantation report-2019; focus theme: Donor and recipient size match. J. Heart Lung Transplant..

[8] Kim S.T., Helmers M.R., Iyengar A., Smood B., Herbst D.A., Patrick W.L., Han J.J., Altshuler P., Atluri P. (2021). Assessing predicted heart mass size matching in obese heart transplant recipients. J. Heart Lung Transplant..

[9] Garg S., Truby L., DeFilippis E., Wu E., Topkara V., Peltz M., Drazner M., Bello N., Farr M. (2022). Association of Predicted Heart Mass from Mesa and Left Ventricular Mass from Dallas Heart Study with Heart Transplant Outcomes in the New Allocation System. J. Heart Lung Transplant..

[10] Chouairi F., Milner A., Sen S., Guha A., Stewart J., Jastreboff A.M., Mori M., Clark K.A., Miller P.E., Fuery M.A. (2021). Impact of Obesity on Heart Transplantation Outcomes. J. Am. Heart Assoc..

[11] Khush K.K., Malinoski D., Luikart H., Wayda B., Groat T., Nguyen J., Belcher J., Nieto J., Neidlinger N., Salehi A. (2023). Left ventricular dysfunction associated with brain death: Results from the Donor Heart Study. Circulation.

[12] O’Donnell C., Tapaskar N., Sanchez P.A., Wayda B., Santana E.J., Pulgrossi R.C., Steffner K., Zhang S., Weng Y., Sun L.Y. (2025). The Association of Echocardiographically Measured Donor Left Ventricular Mass and 1-Year Outcomes After Heart Transplantation. JACC Heart Fail..

[13] Lang R.M., Badano L.P., Mor-Avi V., Afilalo J., Armstrong A., Ernande L., Flachskampf F.A., Foster E., Goldstein S.A., Kuznetsova T. (2015). Recommendations for cardiac chamber quantification by echocardiography in adults: An update from the American Society of Echocardiography and the European Association of Cardiovascular Imaging. J. Am. Soc. Echocardiogr..

[14] Kawel-Boehm N., Kronmal R., Eng J., Folsom A., Burke G., Carr J.J., Shea S., Lima J.A.C., Bluemke D.A. (2019). Left ventricular mass at mri and long-term risk of cardiovascular events: The Multi-Ethnic Study of Atherosclerosis (MESA). Radiology.

